# Integrating Super-Oxidised Solutions into Multimodal Strategies for Orthopaedic Infection Prevention and Management

**DOI:** 10.7759/cureus.96459

**Published:** 2025-11-09

**Authors:** Ioannis S Vasios, Konstantinos G Makiev, Anthimos Keskinis, Paraskevas Georgoulas, Efthymios Iliopoulos, Konstantinos Tilkeridis

**Affiliations:** 1 Department of Orthopaedics and Traumatology, University General Hospital of Alexandroupolis, Democritus University of Thrace, Alexandroupolis, GRC

**Keywords:** dwell time, fracture-related infections (fris), instillation, negative pressure wound therapy (npwt), peri-prosthetic joint infections (pjis), super-oxidised solutions (sos)

## Abstract

Fracture-related infections (FRIs) and periprosthetic joint infections (PJIs) remain among the most challenging complications in orthopaedic surgery, often requiring prolonged and multidisciplinary management. Standard approaches-such as surgical debridement, implant retention or removal, and systemic antibiotic therapy-may be limited in effectiveness, particularly in patients with significant comorbidities or high surgical risk.

This study explores the adjunctive use of super-oxidised solutions (SOS) within existing treatment protocols for orthopaedic infections. It provides a comprehensive overview of their practical application, including intraoperative lavage, postoperative wound care, and integration with negative pressure wound therapy with instillation (NPWT-i). Importantly, this work introduces a structured treatment algorithm designed to guide clinicians in the systematic use of SOS as part of multimodal infection management strategies.

By synthesising available preclinical and clinical data, the study aims to bridge the gap between theoretical understanding and clinical practice. SOS are presented as complementary tools intended to support, not replace, surgical intervention and systemic antimicrobial therapy. Their use may offer additional support in complex cases, particularly in patients for whom traditional approaches alone may be insufficient.

This framework is intended to assist clinicians in decision-making and protocol development, with the goal of improving consistency, safety, and outcomes in the management of FRIs and PJIs.

## Introduction and background

Introduction

Fracture-related infections (FRIs) and periprosthetic joint infections (PJIs) represent two of the most complex and burdensome complications in orthopaedic surgery. These infections not only lead to prolonged morbidity and repeated surgical interventions but are also associated with significant economic and psychosocial consequences. Both are often biofilm-associated, polymicrobial, and complicated by multidrug-resistant organisms, posing formidable treatment challenges [1,2].

Conventional management strategies involve a combination of surgical debridement, removal of infected implants or hardware, and prolonged systemic antibiotic therapy. However, these approaches have limitations. Systemic antibiotics often fail to penetrate avascular biofilms that coat prosthetic surfaces and necrotic bone, limiting their effectiveness [3]. Moreover, elderly or medically frail patients may not tolerate multiple surgeries, and certain resistant pathogens such as Pseudomonas aeruginosa or MRSA reduce the efficacy of conventional antibiotic regimens [4,5].

To address these limitations, adjunctive use of topical antiseptic agents has gained increasing interest. Super-oxidised solutions (SOS) are composed primarily of hypochlorous acid and other reactive oxygen species (ROS). They possess broad-spectrum antimicrobial activity and have demonstrated low cytotoxicity to host tissues [6,7]. Unlike traditional antiseptics such as povidone-iodine or chlorhexidine, SOS do not induce tissue irritation or impair wound healing, making it suitable for use in deep surgical sites and around implants.

This narrative review synthesises current evidence on SOS as adjuncts in FRI and PJI management. We discuss their physical and chemical properties, antimicrobial and antibiofilm mechanisms, preclinical and clinical data, and emerging delivery strategies such as negative pressure wound therapy with instillation (NPWT-i). Importantly, this work also introduces a proposed treatment algorithm that provides step-by-step guidance for integrating SOS into multimodal management strategies. The algorithm outlines their role during intraoperative lavage, postoperative wound care, and in conjunction with NPWT-i, offering clinicians a structured framework to optimise infection control. Our goal is to provide a critical as well as a practical appraisal of how SOS can be incorporated into standard surgical and antibiotic regimens without exaggerating their capabilities.

Materials and methods

This is a narrative review of super-oxidised solutions (SOS) in orthopaedic and wound care, fracture-related infection (FRI), periprosthetic joint infection (PJI), routine wound irrigation, negative-pressure wound-therapy with instillation (NPWT-i) and other topical applications. Given the heterogeneity of available studies and outcome measures, a meta-analysis or pooled quantitative synthesis was not feasible.

Search Strategy

PubMed and Embase were searched from inception to 31 March 2025 with the key terms ("super-oxidised" OR "hypochlorous") AND ("fracture-related infection" OR "periprosthetic joint infection" OR "implant infection"). Reference lists of all full-text articles were screened for additional studies.

Eligibility

English-language, laboratory, animal or human studies that evaluated SOS as irrigation, instillation or topical therapy for musculoskeletal infection were included; abstract-only reports and non-orthopaedic indications were excluded.

Selection and Synthesis

Two authors independently screened titles and abstracts, assessed full texts, and resolved disagreements by consensus. Owing to the heterogeneity of study designs, results were collated narratively and integrated into the relevant sections of this review rather than pooled quantitatively.

## Review

Overview of SOS

Mechanisms of Action of SOS

SOS are produced through the electrolysis of water and sodium chloride, generating ROS, primarily hypochlorous acid (HOCl), that act as the main antimicrobial agents. HOCl is also a natural component of the innate immune response. However, SOS formulations vary among commercial products in terms of concentration, pH, and electrolysis conditions, which can influence antimicrobial efficacy and tissue compatibility [8]. Despite this variability, SOS exert their antimicrobial action through the following mechanisms.

Membrane disruption: ROS destabilise the lipid bilayer of microbial cell membranes, increasing permeability and leading to cell lysis.

Protein oxidation: Hypochlorous acid denatures key bacterial enzymes and structural proteins, impairing function and metabolic processes.

DNA damage and biofilm interference: At sufficient concentrations, ROS can oxidise nucleic acids and impair quorum sensing, thereby inhibiting biofilm formation and promoting biofilm disaggregation [9].

Clinical Properties of SOS

SOS are distinguished from older antiseptics by their low cytotoxicity, nearly-neutral pH (6.2-7.8), and high tissue compatibility, making them especially suitable for use in deep wounds, over implants, or in immunocompromised patients where tissue preservation is essential [7,10]. Unlike agents such as povidone-iodine and hydrogen peroxide, which can impair wound healing by damaging fibroblasts and keratinocytes [11], SOS are stable at room temperature for up to 12 months when unopened, immediately ready for use without dilution, and non-irritant to skin, mucosa, and eyes, as demonstrated in both preclinical and human trials [6,12].

Benefits

SOS exhibit rapid, broad-spectrum microbicidal activity against Gram-positive cocci, Gram-negative bacilli, anaerobes, *Candida *spp. and selected viruses, making them valuable when cultures are negative or polymicrobial [4,10,13]. Because they have a near-neutral pH, SOS are gentle on fibroblasts and keratinocytes, avoiding wound-healing delays often caused by hydrogen peroxide or iodine. In comparative studies, gels containing SOS caused little inflammatory cytokine release and kept cells alive [7,11]. While they do not completely remove established biofilms, SOS can break down the early extracellular matrix, reduce immature biofilms, and improve antibiotic penetration when used with mechanical cleaning [14]. With a 12-month shelf life when unopened, a near-neutral pH (6.2-7.8), and packaging that is ready to use or compatible with NPWT-i systems, SOS are well-suited for emergency care, outpatient clinics, and rural healthcare settings [6,13]. Finally, topical application offers a minimally invasive adjunct for frail, elderly or multimorbid patients who cannot tolerate extensive surgery or aggressive systemic regimens, as documented in diabetic foot and superficial orthopaedic infections [12,15] (Table 1).

**Table 1:** Benefits of super-oxidised solutions (SOS) DAIR: debridement-antibiotics-irrigation-retention, FRI: fracture-related infection, MDR: multi-drug resistant [6-16]

Benefit	Practical implication
Broad-spectrum antimicrobial activity	Gram-positive, Gram-negative, anaerobic, fungal, and some viral pathogens, including multidrug-resistant organisms. Useful for empiric coverage, especially in culture-negative or polymicrobial infections.
Low cytotoxicity, near-neutral pH	Allows ample, repeated lavage without harming fibroblasts or delaying healing (good tissue compatibility).
Early biofilm disruption and improved antibiotic penetration	Disrupts early biofilms and reduces immature biofilm mass during DAIR or initial FRI debridement.
Ready-to-use, room-temperature stability (≤ 12 mo unopened)	Suits emergency, outpatient and resource-limited settings.
No known promotion of antimicrobial resistance	Safe adjunct even in MDR environments.
Minimally invasive option for frail/comorbid patients	Daily rinses reduce bioburden where re-operation is risky.

Risks and Limitations

Despite their potent antimicrobial action, SOS cannot guarantee sterility in deep or complex wounds. Lesman et al. demonstrated that 34 % of anterior cruciate ligament (ACL) grafts remained culture-positive after SOS treatment, underscoring their role as microbial load-reducers rather than stand-alone sterilants [17]. Penetration into mature, mineralised biofilms is also limited, as dense extracellular matrices can shield bacteria even after aggressive chemical and mechanical debridement; this was shown in studies where Staphylococcus aureus persisted following two-stage revision procedures [9,18]. Furthermore, long-term orthopaedic evidence remains scarce, relying largely on pilot studies and case series rather than robust, prospective randomised controlled trials, leaving the durability of infection clearance and implant survival uncertain [14,19]. The easy availability and broad antimicrobial coverage of SOS also carry the risk of over-reliance, where irrigation might be substituted for essential measures such as implant exchange, systemic antibiotic therapy, or flap coverage, thereby allowing chronic infection to smoulder [3].

The evidence regarding the effects of SOS on articular cartilage is similarly indirect. No studies have directly examined SOS exposure on isolated chondrocytes with respect to viability, matrix synthesis, or oxidative damage. Therefore, a blanket claim that SOS are either “safe” or “cytotoxic” to cartilage is not supported. What can be stated with confidence is that SOS demonstrate high compatibility with periarticular soft tissues in routine clinical use, but for native articular cartilage, the safety profile remains uncertain and is likely exposure-time-dependent [7,10,20].

Finally, once bottles are opened, free-chlorine levels decline below bactericidal thresholds within approximately 30 days, so improper storage or reuse may result in irrigation with sub-therapeutic solution [6,13].

These considerations should be taken into account when interpreting the data summarised in Table 2.

**Table 2:** Limitations and risks of super-oxidised solutions (SOS) PJIs: peri-prosthetic joint infections, FRIs: fracture-related infections [9-11,16,20-22]

Limitation	Clinical consequence
Cannot sterilise deep or complex wounds on its own	Still need for thorough debridement and systemic antibiotics
Limited penetration into mature, mineralised biofilm	Chronic PJIs/FRIs often require hardware removal or staged revision
Activity declines ~30 days after bottle opened	Must use single-use vials or date-stamp containers
Avoid direct contact with articular cartilage designated for preservation	Potential injury of chondrocytes

Topical antiseptics as adjuncts to orthopaedic infection management

SOS complement systemic antibiotics by rapidly reducing local bacterial load, disrupting early biofilms, and creating a wound environment conducive to host immune activity. When used intraoperatively as an irrigation fluid or postoperatively through soaked dressings or in conjunction with NPWT, they can access anatomical niches that intravenous agents cannot, including trabecular bone, dead spaces, and implant interfaces [21,23]. Their utility has been demonstrated in both FRIs and PJIs, conditions where biofilm formation, devascularised tissues, and the presence of hardware severely limit the efficacy of systemic antibiotics, and they are increasingly being applied across a broader spectrum of orthopaedic and wound-care infection scenarios. However, SOS should be regarded strictly as an adjunct, not a substitute, for systemic antibiotic therapy or appropriate implant management because both FRIs and PJIs rarely resolve without aggressive intravenous therapy and definitive surgical intervention [3]. Over-reliance on topical irrigation risks leaving residual biofilm, which may lead to persistent or recurrent infection; recent evidence underscores that thorough source control and targeted systemic antibiotics remain the cornerstone of eradication [4,19] (Table 3).

**Table 3:** Clinical applications of super-oxidised solutions (SOS) in orthopaedic infection management DAIR: debridement-antibiotics-irrigation-retention, NPWT-i: negative pressure wound therapy with instillation, DFU: diabetic foot ulcer, FRI: fracture-related infection, PJI: periprosthetic joint infection [10,12-16,19,20,22-26]

Setting	Mode of use	Typical regimen	Key outcomes
Early FRI	High-volume intra-op lavage	250-500 mL per pocket; 2-6L in large cavities; dwell 1-3 min	Decreased bacterial load and faster granulation
Early PJI (DAIR)	Lavage after mechanical debridement	2-6L before closure; dwell 1-3 min	Higher prosthesis-salvage rates vs saline alone
NPWT-i	Instillate fluid for complex wounds	10-20-minute dwell time every 2-4h with -125 mmHg negative pressure	Decrease bacterial load and faster closure
High-risk post-op wounds	Daily rinses / soaked gauze	Once daily until drainage resolves	decrease infection, shorter healing time in DFU; no excess cytokine response

Fracture-Related Infections (FRIs)

Fractures, particularly open or highly comminuted injuries, are characterised by poor vascularity and early biofilm formation on fixation devices. In FRI cases, early microbial control is critical to prevent hardware failure and progression to osteomyelitis. High-volume SOS lavage at the time of initial or revision debridement reduces planktonic bacterial counts and weakens nascent biofilms, thereby complementing systemic therapy that alone penetrates bone poorly [1,3]. Postoperatively, SOS can be applied via NPWT-i or SOS-soaked gauzes since such protocols can achieve faster granulation, smaller wound dimensions, and fewer reoperations [20,23,27]. As shown by Seiser et al., SOS formulations do not provoke a pro-inflammatory cytokine surge, supporting their tissue compatibility [7]. Laboratory data also confirm that SOS possess anti-biofilm activity against planktonic bacteria and immature biofilms [14]. However, mature biofilms remain more difficult to eradicate; thus, irrigation with SOS after debridement can hinder their re-establishment and enhance antibiotic penetration by disrupting the extracellular matrix [9]. Importantly, this local oxidative mechanism does not appear to promote antibiotic-resistant organisms [13,17].

Periprosthetic Joint Infections (PJIs)

Early (< 4 weeks) PJIs are usually managed with debridement, antibiotics and implant retention (DAIR). In this setting, 2-6L of SOS after meticulous mechanical cleaning reduces residual pathogen load without increasing cytotoxicity, thereby improving the likelihood of prosthesis salvage [16,22,27]. Due to the fact that SOS act through oxidation rather than classical antibiotic pathways, they offer broad empiric coverage in culture-negative cases and retain activity against multidrug-resistant organisms [4]. The same property makes them attractive, where vancomycin-loaded spacers have proved inadequate against resistant *Pseudomonas aeruginosa* [5]. For extensive soft-tissue defects, combining SOS with NPWT maintains a moist, oxygen-rich environment that encourages granulation while keeping local bacterial counts low [15,28].

Surgical Irrigation During Debridement

Radical mechanical debridement must be paired with generous fluid lavage to minimise residual biofilm and prevent reseeding. Data from observational studies indicate that the volume of irrigation is an independent predictor of DAIR success in PJIs [16,22,27]. SOS are well-suited for this step since, in comparative bench testing, they resulted in significantly higher bacteria clearance than povidone-iodine or chlorhexidine, while at the same time they were able to preserve fibroblast viability [10,13]. Because of their low cytotoxicity and broad antimicrobial range, SOS can safely be used in high volumes (at least 250-500 mL per contaminated pocket and, in large cavities or arthroplasties, 2-6L in total) during initial FRI debridement or DAIR for early PJI. The solution is typically allowed to dwell for 1-3 min before suction and can be repeated until outflow runs clear, providing both mechanical and chemical bioburden reduction without impairing wound healing. As previously noted, evidence on the safety and cytotoxicity of SOS for articular cartilage is limited. Accordingly, prolonged exposure should be avoided. Based on current data and expert consensus regarding oxidising antiseptics, exposure times should generally not exceed about five minutes unless the solution is diluted (the precise safe dilution still remains undefined). Therefore, for intra-articular application in native joints, brief rinsing or irrigation followed by immediate removal is advised rather than prolonged soaking or dwell use [6,7,10,15,21].

Postoperative wound care in patients at high risk for re-infection

Postoperative wound infections may develop in patients with diabetes, peripheral vascular disease, or prior wound complications. As shown by Aragón-Sánchez et al., postoperative use of SOS not only reduced infection but also shortened healing time [12]. Supporting its safety profile, Seiser et al. reported that SOS did not induce excessive inflammatory responses in human skin models, indicating suitability for prolonged use [7]. As a consequence, these patients benefit from wound management strategies that minimise microbial colonisation without inhibiting healing. Thus, SOS can be applied (a) as a wound rinse during dressing changes, (b) via soaked gauzes or sponges in surgical drains and (c) through daily instillation in high-risk sites.

In the authors’ current practice, SOS is applied daily to surgical sites with persistent drainage, high microbial burden, or delayed healing, providing a practical adjunct to promote recovery in challenging cases. This treatment strategy is particularly useful for patients unable to tolerate further surgery.

Clinical protocols

Supplementation to NPWT-Instillation (NPWT-i)

Beyond standard NPWT, the addition of instillation (NPWT-i) is indicated when the clinical priority is active wound cleansing and bioburden control. Typical scenarios include contaminated or infected wounds, wounds with heavy bioburden, difficult-to-granulate beds, or those producing thick/viscous exudate that standard NPWT struggles to remove. NPWT-i also has a role when sharp debridement is delayed or unavailable, or when non-viable tissue persists after debridement, particularly with through-hole foams designed to lift debris [24,27,29] (Table 4).

**Table 4:** Indications for NPWT-i NPWT-i: negative pressure wound therapy with instillation [21-23,26,28,30-33]

Indication	Evidence
Contaminated or infected wounds	Provides wound cleansing, reduces bacterial bioburden more effectively than NPWT alone.
Heavy or viscous exudate difficult to remove by suction alone	Instillation helps dissolve and clear thick exudates.
Wounds with persistent biofilm or non-viable tissue after debridement	NPWT-i with through-hole foams can disrupt debris and biofilm.
Difficult-to-granulate wounds or stalled healing on NPWT	Escalation option when NPWT alone is insufficient.
Immediate post-operative use in contaminated wounds (after debridement)	Early initiation reduces bacterial load and promotes healing.
Delayed or unavailable repeated surgical/sharp debridement	NPWT-i serves as an adjunct for interim wound cleansing.
Complex orthoplastic and traumatic wounds	Improved closure rates, fewer complications compared to NPWT alone.

Current evidence demonstrates that NPWT-i improves primary closure rates and reduces complications compared with NPWT or standard care in orthoplastic and heterogeneous wound populations, supporting its use in complex wounds where bacterial burden and cleansing are the main barriers to progress [32,33]. Mechanistic data further show that NPWT-i disrupts biofilm structure and, with appropriate solutions, reduces viable organisms more effectively than NPWT alone [31]. Clinical data reinforce these findings: de Angelis et al. reported reduced bacterial load and improved wound closure rates in infected surgical sites, while Armstrong et al. demonstrated enhanced healing and lower reinfection rates in post-amputation wounds [15,23].

Timing of NPWT-i is flexible but often favours early initiation. It can be applied immediately post-operatively after adequate debridement when contamination, infection, or heavy exudation is anticipated. In fact, randomised data demonstrate a greater reduction in bacterial load by the first dressing change when NPWT-i was allocated intra-operatively compared with NPWT alone [30]. Conversely, escalation to NPWT-i is also appropriate if a wound on standard NPWT remains highly contaminated, exudative, or stalled in granulation, reflecting a shift in consensus that NPWT-i is no longer only a salvage option but a proactive therapy for wounds not responding adequately to NPWT [16,27,33].

SOS, when combined with NPWT-i, enhances outcomes by continuously irrigating the wound bed, disrupting early biofilm formation, and promoting granulation tissue development. As shown by Kim et al. and de Angelis et al., in current practice, complex orthopaedic wounds are treated with NPWT-i using SOS with a dwell time of 10-15 minutes every 2-4 hours, a regimen adapted from their favourable results [15,30].

Contraindications and limitations must also be respected. NPWT-i should not be used over exposed, unprotected organs or vessels, in undrained abscesses, acutely ischemic wounds, or as a bolster over skin grafts or dermal substitutes. Therapy is also unsuitable where maintaining a reliable seal is impossible, in cases where dry eschar entirely overlies the wound prior to debridement, or when the wound is very small [29]. Additional exclusions, drawn from pragmatic trial criteria and manufacturer-aligned guidance, include malignancy in the wound, untreated osteomyelitis, unexplored fistulas, thoracic or abdominal cavity instillation, poorly perfused ischemic limbs, or unprotected vascular/nerve/anastomotic structures. Ensuring hemostasis is essential, particularly in anticoagulated patients, before immediate post-operative application [29,31] (Table 5).

**Table 5:** Contraindications for NPWT-i NPWT-i: negative pressure wound therapy with instillation [22,23,31,32]

Contraindication	Consideration
Exposed, unprotected organs, vessels, anastomoses, or nerves	Risk of erosion, bleeding, or damage
Undrained abscess	Must be surgically drained before NPWT-i
Acute limb ischemia or poorly perfused ischemic tissue	Lack of healing potential, risk of necrosis
100% dry eschar (pre-debridement)	Ineffective unless debridement performed
Very small wounds where seal cannot be maintained	Therapy ineffective without airtight seal
Malignancy in wound bed	Theoretical risk of tumor spread
Untreated osteomyelitis	Infection must be treated before NPWT-i
Unexplored fistulas	Risk of instillation fluid entering cavities
Thoracic or abdominal cavity wounds	Contraindicated due to risk of organ damage
Post-graft or dermal substitute bolster	May impair graft take
Inadequate hemostasis (esp. anticoagulated patients)	Risk of bleeding post-operatively

**Figure 1:**
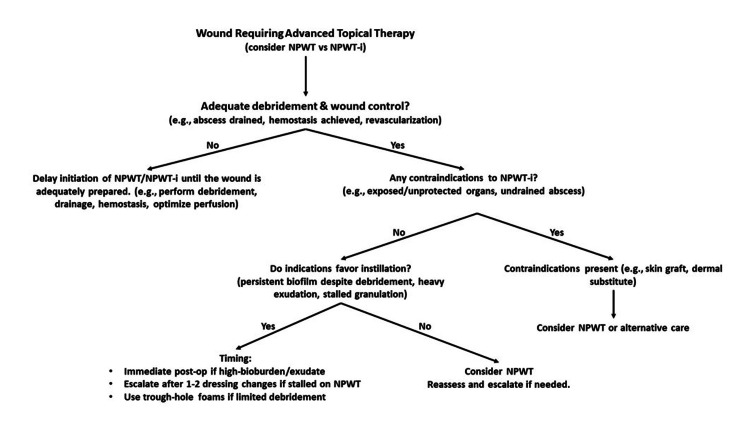
Decision algorithm for NPWT vs NPWT-i Decision algorithm for NPWT versus NPWT-i. NPWT-i is considered following adequate debridement and wound control, unless contraindicated. Instillation is favoured for wounds with high bioburden, heavy exudation, or stalled granulation, while NPWT alone or alternative care is used when these criteria are not met. NPWT-i: negative pressure wound therapy with instillation [16,20-24,29-33]

In summary, NPWT-i may be initiated immediately following debridement in contaminated, infected, or heavily exudative wounds, and as an escalation when NPWT alone fails to control exudate, reduce bioburden, or stimulate granulation (Figure 1).

When combined with appropriate instillation solutions such as SOS, NPWT-i offers enhanced wound cleansing, biofilm disruption, and improved healing trajectories, while remaining part of an integrated wound management plan that prioritises debridement, antibiotics when indicated, and thorough wound bed optimisation. A summary of representative clinical studies evaluating NPWT-i with super-oxidised and other antiseptic solutions is provided in Table 6.

**Table 6:** Summary of clinical studies evaluating NPWT-i with various super-oxidised solutions (SOS) and other agents NR: not reported, LOS: length of hospital stay, NPWT-i: negative pressure wound therapy with instillation, RCT: randomised control trial, PHMB: polyhexanide (polyhexamethylene biguanide), PVA: polyvinyl alcohol [10,12,15,28-30,32,33]

Author	Type of study	Indication	Pts	Healing time	Outcomes	Adverse events	Solution used	Setting
Zhao et al. (2024) [28]	Retrospective Study	Diabetic foot infections	134	Exact values NR	No difference in wound-bed prep time; LOS similar (NR exact values)	NR	Prontosan® vs Normal Saline	NPWT-i: dwell 10–15 min; 4 cycles/day; continuous -125 mmHg; system change every 4-6 days
De Pellegrin et al. (2023) [33]	Systematic review & meta-analysis	Orthoplastic / trauma wounds	871	NR (directionally favoured NPWT-i)	No difference in number of surgical procedures to final healing; Higher primary wound-closure rate with NPWT-i	Lower complication rates with NPWT-i	Normal saline, Microdacyn®	NPWT-i: dwell 10-20 min; cycles every 3-4 h
Kanapathy et al. (2020) [32]	Systematic review & meta-analysis	Mixed adult wounds of varied aetiology treated	542 pts 624 wounds	Mean time to wound healing ≈ 8.49 days; mean therapy duration 10.69 days	Complete healing in 93.65%	No severe adverse effects reported.	Mostly normal saline; some Microdacyn®/Dermacyn®	NPWT-i: dwell 14.23 min; cycle every 4.2 h; duration 10.69 days; -125 mmHg
Kim et al. (2020) [30]	Multicenter RCT	Operatively debrided wounds	132	Time to readiness for closure similar between groups	No significant difference in number of inpatient OR debridements	Re-hospitalisation risk 3.1× higher with standard NPWT; complications similar	Prontosan®	NPWT-i: dwell 10 min; cycle every 3.5 h; -125 mmHg
Aragón-Sánchez et al. (2013) [12]	Prospective case series	Complicated diabetic foot osteomyelitis post-op wounds with suspected residual infected bone	14	Median 6.8 weeks to healing	Limb salvage 100%	No side effects. Neither allergies nor skin dermatitis were found.	Dermacyn®	Rinsed into post-op cavities during dressing changes; no instillation
de Angelis et al. (2012) [15]	Case report	Stage IV sacral pressure ulcer in paraplegic patient	1	full closure time NR	Lower exudate within 48h; disappearance after five applications	NR	Dermacyn®	15-20 min topical soak per dressing; followed by NPWT (-80 mmHg); no instillation
Piaggesi et al. (2010) [10]	RCT	post-surgical diabetic foot lesions	40	Mean 10.5 ± 5.9 wks with Dermacyn vs 16.5 ± 7.1 wks control	4 re-interventions (Dermacyn) vs 11 control over 6 mo; Healing at 6 mo: 90% Dermacyn vs 55% control	Overall adverse effects similar; reinfections significantly fewer with Dermacyn	Dermacyn®	Topical twice daily; no instillation; wounds left open to heal by secondary intention
Timmers et al. (2009) [29]	Retrospective case-control cohort	Post-traumatic osteomyelitis after debridement	124	Shorter total LOS; faster course vs controls (exact days NR)	Fewer procedures; infection recurrence 10% vs 58.5% in controls; Remission rate 90% in instillation group	NR	PHMB	NPWT-i: PVA foam at ≥300 mmHg; irrigated 3×/day with PHMB solution

Proposed Treatment Algorithm for NPWT-i in FRI and PJI Management

Management of FRIs and PJIs begins with thorough clinical assessment and prompt surgical debridement, followed by high-volume lavage using SOS. This is complemented by appropriate systemic antibiotic therapy tailored to microbiological findings.

As an adjunctive measure, NPWT-i may be applied to optimise infection control and wound bed preparation. NPWT-i is administered with continuous negative pressure at -125 mmHg, using SOS as the instillation solution. As cited in expert consensus guidelines, the utilisation of SOS is preferred over polyhexanide-based solutions due to their broad-spectrum antimicrobial activity, low cytotoxicity, and favourable safety profile.

The instillation volume should be sufficient to fully saturate the wound foam, adjusted according to wound dimensions and exudate levels. Each instillation cycle includes a 15-minute dwell time every two hours, consistent with current consensus recommendations suggesting 10-20 minutes as optimal for effective biofilm disruption.

NPWT-i therapy is continued until infection control is demonstrated by two consecutive negative tissue cultures, after which NPWT-i can be discontinued to proceed with definitive wound closure and reconstruction. Ongoing monitoring through clinical assessment and microbiological evaluation is essential throughout the treatment process, as presented in the following section.

**Figure 2:**
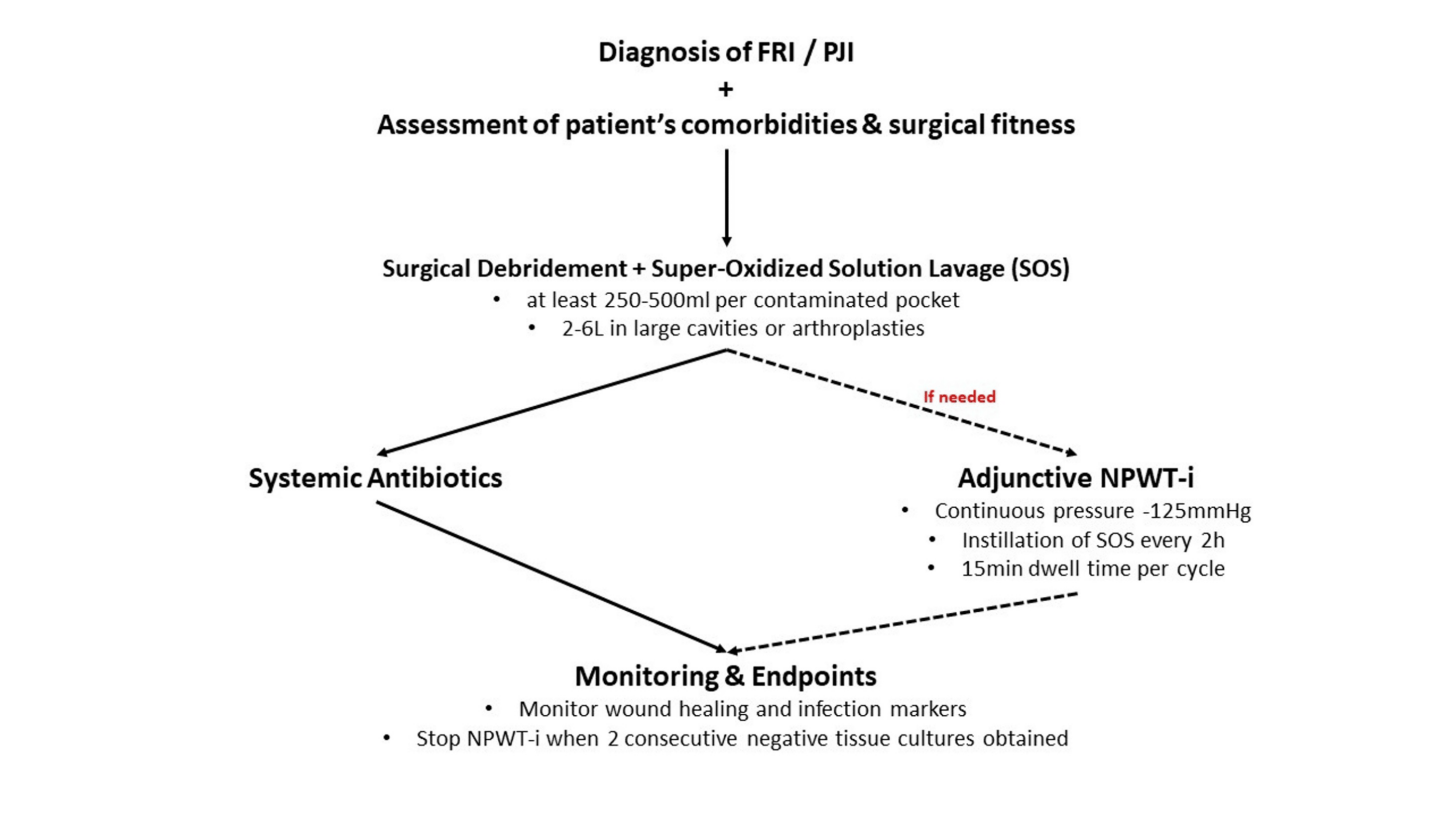
Proposed treatment protocol Proposed treatment protocol for fracture-related infections (FRIs) and preprosthetic joint infections (PJIs) using super-oxidised solutions (SOS) and negative pressure wound therapy with instillation (NPWT-i). Management begins with assessment and surgical debridement plus high-volume SOS lavage, followed by systemic antibiotics. Adjunctive NPWT-i may be added with continuous -125 mmHg pressure and SOS instillation (15 min dwell every 2 hours). Therapy is monitored with clinical and microbiological endpoints, discontinuing NPWT-i once two consecutive negative tissue cultures are achieved. [1-3,6,10,12,15-20,22,24,25,27-33]

This proposed algorithm provides a conceptual framework for the integration of SOS and NPWT-i in the management of FRIs and PJIs. It is based on current evidence and expert interpretation but has not yet been clinically validated as a standardised treatment guideline. Taking all the above into consideration, we demonstrate the following key points to support effective integration of SOS into everyday clinical practice (Figure 2).

Follow-Up and Outcome Monitoring After Treatment of FRIs and PJIs

The follow-up of patients with FRI and PJI is essential to ensure eradication of infection, promote bone healing, and restore function. According to international consensus recommendations, follow-up should be structured and multidisciplinary, focusing on clinical, laboratory, and radiological evaluation over time. Early reviews at two to six weeks aim to assess wound healing, infection control, and antibiotic tolerance, while subsequent assessments at three, six, and 12 months monitor fracture consolidation, implant stability, prosthesis position, bone integrity and functional recovery. Clinical evaluation should confirm the absence of local infection signs such as redness, pain, or drainage, as well as stable fixation with good soft-tissue coverage. Laboratory monitoring, particularly serial C-reactive protein (CRP) measurements, supports early detection of recurrence, whereas imaging (plain radiographs routinely, whilst CT, MRI, and nuclear scans if recurrence or loosening is suspected) aids in assessing bone healing and/or implant stability. Successful treatment of FRIs and PJIs is defined by three main endpoints: (a) infection eradication (no clinical, laboratory, or radiological evidence of infection and no need for further antibiotic or surgical intervention), (b) fracture healing (radiological consolidation without mechanical instability) and (c) functional recovery.

A clinically successful outcome requires the absence of infection recurrence at least 12 months after completion of therapy. Indicators of treatment failure include persistent drainage, rising inflammatory markers, implant loosening, or nonunion. These principles are supported by the International FRI Consensus Group and related expert publications [1,3,34-38].

## Conclusions

Super-oxidised solutions (SOS) represent a promising adjunct in the management of fracture-related infections (FRIs) and periprosthetic joint infections (PJIs). Their integration alongside surgical debridement, systemic antibiotics, and NPWT-i appears to enhance wound healing and infection control in selected patient populations. However, SOS should complement (not replace) established surgical and antimicrobial protocols. Future research should prioritise high-quality randomised controlled trials comparing SOS with debridement, antibiotics, and implant retention (DAIR) versus standard care using saline irrigation. Key endpoints should include infection eradication, implant survival, wound healing time, and adverse events. Further studies on optimal dosing, application regimens, and cost-effectiveness are warranted to define the clinical role of SOS in musculoskeletal infections.

## References

[1] Metsemakers WJ, Morgenstern M, McNally MA (2018). Fracture-related infection: a consensus on definition from an international expert group. Injury.

[2] Gbejuade HO, Lovering AM, Webb JC (2015). The role of microbial biofilms in prosthetic joint infections. Acta Orthop.

[3] Zimmerli W, Sendi P (2017). Orthopaedic biofilm infections. APMIS.

[4] Goswami K, Parvizi J (2020). Culture-negative periprosthetic joint infection: is there a diagnostic role for next-generation sequencing?. Expert Rev Mol Diagn.

[5] Chang MJ, Song MK, Shin JH, Yoon C, Chang CB, Kang SB (2019). Two-stage approach to total knee arthroplasty using colistin-loaded articulating cement spacer for vancomycin-resistant Pseudomonas aeruginosa infection in an arthritic knee. Eur J Orthop Surg Traumatol.

[6] Steenvoorde P, van Doorn LP, Jacobi CE, Oskam J (2007). An unexpected effect of Dermacyn on infected leg ulcers. J Wound Care.

[7] Seiser S, Cerbu D, Gallhofer A, Matiasek J, Elbe-Bürger A (2022). Comparative assessment of commercially available wound gels in ex vivo human skin reveals major differences in immune response-modulatory effects. Sci Rep.

[8] Winterbourn CC, Kettle AJ (2013). Redox reactions and microbial killing in the neutrophil phagosome. Antioxid Redox Signal.

[9] Koo H, Allan RN, Howlin RP, Stoodley P, Hall-Stoodley L (2017). Targeting microbial biofilms: current and prospective therapeutic strategies. Nat Rev Microbiol.

[10] Piaggesi A, Goretti C, Mazzurco S (2010). A randomized controlled trial to examine the efficacy and safety of a new super-oxidized solution for the management of wide postsurgical lesions of the diabetic foot. Int J Low Extrem Wounds.

[11] Lineaweaver W, Howard R, Soucy D (1985). Topical antimicrobial toxicity. Arch Surg.

[12] Aragón-Sánchez J, Lázaro-Martínez JL, Quintana-Marrero Y, Sanz-Corbalán I, Hernández-Herrero MJ, Cabrera-Galván JJ (2013). Super-oxidized solution (Dermacyn Wound Care) as adjuvant treatment in the postoperative management of complicated diabetic foot osteomyelitis: preliminary experience in a specialized department. Int J Low Extrem Wounds.

[13] Kaehn K (2007). Dermacyn on the infected foot ulcers of 10 patients. J Wound Care.

[14] Alves PJ, Barreto RT, Barrois BM, Gryson LG, Meaume S, Monstrey SJ (2021). Update on the role of antiseptics in the management of chronic wounds with critical colonisation and/or biofilm. Int Wound J.

[15] de Angelis B, Lucarini L, Agovino A (2013). Combined use of super-oxidised solution with negative pressure for the treatment of pressure ulcers: case report. Int Wound J.

[16] González-Alonso M, Guerra-González A, Villar-Suárez V, Fernández-Castilla B, Sánchez-Lázaro JA (2025). Efficacy of different irrigation solutions on bacterial biofilm in periprosthetic joint infections: a systematic review and network meta-analysis. Life (Basel).

[17] Lesman J, Nowak K, Poszepczyński J, Compagnoni R, Randelli P, Domżalski M (2025). Effectiveness of a super-oxidized solution for decontaminating ACL grafts: a prospective study. J Orthop Surg Res.

[18] George J, Newman JM, Klika AK, Miller EM, Tan TL, Parvizi J, Higuera CA (2018). Changes in antibiotic susceptibility of Staphylococcus aureus between the stages of 2-stage revision arthroplasty. J Arthroplasty.

[19] Gatti M, Barnini S, Guarracino F (2022). Orthopaedic implant-associated staphylococcal infections: a critical reappraisal of unmet clinical needs associated with the implementation of the best antibiotic choice. Antibiotics (Basel).

[20] Shaik A, Chakrapani AS, Hayat U, Ahmed A, Selvarajan DP, Shaik N (2025). Efficacy of debridement, antibiotics, and implant retention for periprosthetic joint infections following hip and knee arthroplasty: a retrospective cohort study. Cureus.

[21] Saeg F, Schoenbrunner AR, Janis JE (2021). Evidence-based wound irrigation: separating fact from fiction. Plast Reconstr Surg.

[22] Longo UG, De Salvatore S, Bandini B, Lalli A, Barillà B, Budhiparama NC, Lustig S (2024). Debridement, antibiotics, and implant retention (DAIR) for the early prosthetic joint infection of total knee and hip arthroplasties: a systematic review. J ISAKOS.

[23] Armstrong DG, Lavery LA (2005). Negative pressure wound therapy after partial diabetic foot amputation: a multicentre, randomised controlled trial. Lancet.

[24] Back DA, Scheuermann-Poley C, Willy C (2013). Recommendations on negative pressure wound therapy with instillation and antimicrobial solutions - when, where and how to use: what does the evidence show?. Int Wound J.

[25] Kim PJ, Attinger CE, Constantine T (2020). Negative pressure wound therapy with instillation: International consensus guidelines update. Int Wound J.

[26] Gramlich Y, Johnson T, Kemmerer M, Walter G, Hoffmann R, Klug A (2020). Salvage procedure for chronic periprosthetic knee infection: the application of DAIR results in better remission rates and infection-free survivorship when used with topical degradable calcium-based antibiotics. Knee Surg Sports Traumatol Arthrosc.

[27] Kim PJ, Applewhite A, Dardano AN (2018). Use of a novel foam dressing with negative pressure wound therapy and instillation: recommendations and clinical experience. Wounds.

[28] Zhao J, Shi K, Zhang N, Hong L, Yu J (2024). Assessment between antiseptic and normal saline for negative pressure wound therapy with instillation and dwell time in diabetic foot infections. Sci Rep.

[29] Timmers MS, Graafland N, Bernards AT, Nelissen RG, van Dissel JT, Jukema GN (2009). Negative pressure wound treatment with polyvinyl alcohol foam and polyhexanide antiseptic solution instillation in posttraumatic osteomyelitis. Wound Repair Regen.

[30] Kim PJ, Lavery LA, Galiano RD (2020). The impact of negative-pressure wound therapy with instillation on wounds requiring operative debridement: Pilot randomised, controlled trial. Int Wound J.

[31] Tahir S, Malone M, Hu H, Deva A, Vickery K (2018). The effect of negative pressure wound therapy with and without instillation on mature biofilms in vitro. Materials (Basel).

[32] Kanapathy M, Mantelakis A, Khan N, Younis I, Mosahebi A (2020). Clinical application and efficacy of negative pressure wound therapy with instillation and dwell time (NPWTi-d): a systematic review and meta-analysis. Int Wound J.

[33] De Pellegrin L, Feltri P, Filardo G, Candrian C, Harder Y, Galetti K, De Monti M (2023). Effects of negative pressure wound therapy with instillation and dwell time (NPWTi-d) versus NPWT or standard of care in orthoplastic surgery: a systematic review and meta-analysis. Int Wound J.

[34] Rupp M, Walter N, Bärtl S, Heyd R, Hitzenbichler F, Alt V (2024). Fracture-related infection-epidemiology, etiology, diagnosis, prevention, and treatment. Dtsch Arztebl Int.

[35] Metsemakers WJ, Morgenstern M, Senneville E (2020). General treatment principles for fracture-related infection: recommendations from an international expert group. Arch Orthop Trauma Surg.

[36] Al-Jabri T, Ridha M, Wood MJ, Kayani B, Jayadev C, McCulloch RA, Schemitsch E (2024). An overview of the current diagnostic approach to periprosthetic joint infections. Orthop Rev (Pavia).

[37] Guan H, Fu J, Li X (2019). The 2018 new definition of periprosthetic joint infection improves the diagnostic efficiency in the Chinese population. J Orthop Surg Res.

[38] Parvizi J, Tan TL, Goswami K, Higuera C, Della Valle C, Chen AF, Shohat N (2018). The 2018 definition of periprosthetic hip and knee infection: an evidence-based and validated criteria. J Arthroplasty.

